# The Effect of Clinical Knowledge on the Evaluation of Sacroiliac Joint Radiography in Patients with Spondyloarthritis

**DOI:** 10.3390/diagnostics15243093

**Published:** 2025-12-05

**Authors:** Sezgin Zontul, Zeynep Kaya, Mesude Seda Aydoğdu, Ahmet Kadir Arslan, Elif İnanç, Zeynep Maraş Özdemir, Servet Yolbaş

**Affiliations:** 1 Division of Rheumatology, Department of Physical Medicine and Rehabilitation, Inonu University Faculty of Medicine, Malatya 44280, Turkey; 2 Division of Rheumatology, Department of Internal Medicine, Istanbul Training and Research Hospital, İstanbul 34098, Turkey; zeynepkaya00@gmail.com; 3 Division of Rheumatology, Department of Internal Medicine, Malatya Training and Research Hospital, Malatya 44330, Turkey; kinaci_seda@hotmail.com; 4 Department of Biostatistics and Medical Informatics, Inonu University Faculty of Medicine, Malatya 44280, Turkey; arslan.ahmet@inonu.edu.tr; 5 Division of Rheumatology, Department of Internal Medicine, Inonu University Faculty of Medicine, Malatya 44280, Turkey; elif.temelli@hotmail.com (E.İ.); servet.yolbas@inonu.edu.tr (S.Y.); 6 Department of Radiology, Inonu University Faculty of Medicine, Malatya 44280, Turkey; zynpmaras@yahoo.com

**Keywords:** sacroiliitis, spondyloarthritis, radiography, clinical context, interobserver variability

## Abstract

**Background/Objectives:** This study investigates whether a patient’s clinical status influences the evaluation of sacroiliac joint (SIJ) radiographs. **Methods**: The study involved analysing SIJ radiographs of patients diagnosed with spondyloarthritis (SpA) at our clinic. Two rheumatologists working at another centre evaluated the images independently. Three months were allowed to elapse so that the rheumatologists would forget the results of the first evaluation. The radiographs were then re-evaluated in a different order by the same rheumatologists. However, during the second evaluation, the evaluators were also provided with general clinical information about the patients. Inter- and intra-observer agreement were assessed. **Results**: In the first blinded evaluation of our study, we found moderate-to-substantial agreement between rheumatologists (right κ: 0.534; *p* < 0.001; left κ: 0.609; *p* < 0.001) and statistically significant interpretation agreement. In the second evaluation, we observed an increase in agreement (κ increased from 0.534 to 0.774 for the right SIJ and from 0.609 to 0.855 for the left SIJ), and these metrics were also significant. **Conclusions**: The interpretation of SIJ radiographs showed notable inter-observer variability in the absence of clinical information. Incorporating clinical context significantly improved the agreement between readers. As conventional radiography remains central to spondyloarthritis classification, these findings highlight the value of clinical data in enhancing the reliability of radiographic assessment. To our knowledge, this is the first study to systematically demonstrate the impact of clinical information on inter-observer agreement in SIJ radiograph interpretation.

## 1. Introduction

SpA is the currently accepted term for a group of inflammatory disorders characterised by similar genetic and clinical manifestations. The SpA group typically presents with sacroiliitis, spondylitis, peripheral arthritis, enthesitis, dactylitis, psoriasis, uveitis and inflammatory bowel disease (IBD) in varying combinations [1]. The current classification system divides SpA patients into two main categories: axial spondyloarthritis and peripheral spondyloarthritis [2].

Axial spondyloarthritis (axSpA) is a long-term inflammatory condition predominantly involving the axial skeleton. The global prevalence of axSpA ranges from 0.2% to 1.6%, and this figure is largely influenced by the prevalence of HLA-B27 in the general population. The disease typically begins before the age of 45, and although radiographic axSpA is more prevalent in men, the gender distribution is more balanced in non-radiographic forms. AxSpA is also often associated with a delay in diagnosis of several years. The axSpA spectrum includes both radiographic axSpA, which is characterised by structural changes in the SIJ or spine that are observable via conventional radiographs and non-radiographic axSpA in which such imaging findings are absent [3]. Chronic back pain that persists for more than three months and occurs nearly every day is the hallmark symptom. This pain typically begins insidiously, exhibits inflammatory features and is often accompanied by morning stiffness. The discomfort usually centres on the lower back and hip regions [4]. Human Leukocyte Antigen B27 (HLA-B27) positivity is common among patients with axSpA, and it is an important genetic biomarker in terms of prognosis [5].

Conventional radiography and magnetic resonance imaging (MRI) of the SIJ are important in diagnosing and classifying axSpA [6]. One study compared low-dose CT with conventional radiography and found that conventional radiography missed more than half of patients with axSpA. In contrast, low-dose CT missed two-thirds of SIJs with structural changes indicating axSpA. The same study showed that T1-weighted MRI has a similar sensitivity to low-dose CT and a higher sensitivity than radiography in terms of detecting structural lesions, especially erosions and joint space changes, including ankylosis [7]. Although MRI is an important tool for detecting and scoring bone marrow oedema, as well as structural lesions, there has been no final agreement on thresholds in this regard [7,8].

Despite the development and use of advanced imaging techniques, such as MRI and CT (computed tomography), the conventional radiography of the SIJ remains the cornerstone of diagnosing and classifying SpA [9]. However, different rheumatologists may interpret these imaging modalities differently. Even the same rheumatologist may produce different evaluations at different times [10]. One reason for this is that SIJs have a complex three-dimensional configuration and individual anatomical variation [11]. Overlying intestinal gas and soft tissues can make it difficult to detect abnormalities. This has led to various imaging approaches being used in clinical practice to optimise the radiographic evaluation of the SIJ. However, studies in which different radiographic imaging techniques were applied revealed that the level of agreement was not significantly different from that obtained with AP pelvic radiography [12]. A study examining the consistency of SIJ grading by rheumatologists and radiologists revealed significant discrepancies, particularly for grades 1 and 2. Furthermore, extensive training was found to have no impact on readers’ ability to diagnose sacroiliitis [13]. The fact that neither training nor radiographs taken using different protocols solved the problem of evaluating SIJ radiographs suggests that there may be other reasons for the inconsistency between evaluators. Given the importance of clinical information in diagnosing the disease, it could be argued that this leads to the biassed evaluation of SIJ radiographs.

In most SIJ evaluation studies, observers were asked to assess SIJs without considering a patient’s clinical characteristics. Although some studies have involved local rheumatologists with clinical knowledge, there is no direct evidence in the literature of a systematic exploration of how access to clinical knowledge affects the interpretation of the same observers. Rheumatologists may be influenced by a patient’s clinical characteristics (age, gender and genetics) when assessing the SIJ.

This study investigates whether clinical information influences the evaluation of sacroiliac joint radiographs. We did not find any studies in the literature evaluating the re-reading of SIJ graphs after clinical information was provided. In our study, SIJ radiographs were evaluated by two rheumatologists, both with and without clinical information, and both inter-observer and intra-observer consistency were analysed. In this respect, our study offers a unique approach that examines the effect of clinical information on radiographic evaluation in diagnostic processes.

## 2. Materials and Methods

### 2.1. Study Design and Participants

This was a retrospective observer agreement study designed to evaluate the impact of clinical information on the interpretation of SIJ radiographs in patients with SpA. The study was conducted using the SIJ radiographs of patients diagnosed with peripheral or axial SpA at our clinic, using the classification criteria of the Assessment of SpondyloArthritis International Society (ASAS) [14,15]. Ethical approval was obtained for this study by the Inonu University Clinical Research Ethics Committee, with number 2024/6851.

### 2.2. Imaging Procedure

Standard anteroposterior (AP) pelvic radiographs were obtained for all patients to evaluate their SIJs. During imaging, patients were placed in the supine position on the examination table. The anterior superior iliac spines were positioned equidistant from the midline to ensure pelvic symmetry. The upper edge of the cassette was aligned approximately four fingerbreadths (5–7 cm) above the iliac crests. The X-ray beam was angled at 10–25° in a caudocranial direction and centred over the symphysis pubis. This technique was used to optimise the visualisation of the sacroiliac joint planes [13].

### 2.3. Radiographic Evaluation

A radiologist confirmed the presence or absence of SIJ involvement using previously obtained MR images of the joint. Two rheumatologists working at a different centre evaluated the images independently. Those who evaluated the graphs had been working as rheumatology specialists for approximately three years. Radiographic evaluations were performed by rheumatologists based on the modified New York criteria. According to this system, radiographs were classified as follows: grade 0, indicating normal findings; grade I, representing suspicious changes; grade II, showing mild abnormalities, such as small, localised areas of erosion or sclerosis without alteration in joint space; grade III, indicating clear pathological changes, including extensive erosions, visible sclerosis, joint space narrowing or widening or partial ankylosis, and grade IV, denoting complete ankyloses [16]. The radiographs were categorised as either radiographic or non-radiographic by the rheumatologists conducting the evaluation. Radiographic axSpA was defined according to the modified New York criteria, which require at least a bilateral sacroiliitis grade ≥ II or a unilateral sacroiliitis grade ≥ III on conventional radiographs (15). Three months later, the same rheumatologists re-evaluated the radiographs in a different order to avoid being influenced by the previous evaluation results. However, during the second evaluation, the evaluators were also provided with general clinical information about the patients. This included a patient’s age; gender; date of rheumatic disease diagnosis; history of psoriasis, uveitis, and inflammatory bowel disease and HLA-B27 status. For patients whose HLA-B27 testing had not been performed, the clinical information sheet provided to the rheumatologists explicitly stated “Not checked,” ensuring that missing results were identifiable rather than interpreted as absent or negative. Additionally, information was shared regarding low back and hip pain, morning stiffness and its duration, heel pain, C-reactive protein (CRP) levels and sedimentation rates during the active period of the disease. The results were recorded, and the differences in interpretation between and within observers were analysed.

### 2.4. Statistical Analysis

According to the theoretical power analysis findings, at a 5% significance level and an 80% test power, the required sample size to detect a statistically significant difference between groups when using the alternative hypothesis (H1) in a two-way and paired-samples *t*-test is at least 57 in total. The demographic and clinical variables in the dataset are summarised using the relevant statistics. The scoring variable used to assess the rheumatologists’ diagnostic agreement is summarised in a median (first quartile–third quartile) format. A Wilcoxon signed-rank test was used to assess the systematic differences between rheumatologists, and a Cohen’s weighted (linear) and unweighted Kappa analysis was used to analyse the agreement between them. Confidence intervals and *p*-values were calculated using the bootstrap technique with 1000 repetitions for changes in kappa values, with *p* < 0.05 being accepted as the level of statistical significance. The analyses were performed using R software, version 4.1.2 (R Foundation for Statistical Computing, Vienna, Austria) and IBM SPSS Statistics for Windows, version 27.0 (IBM Corp., Armonk, NY, USA). Table 1 summarises how the Kappa analysis is interpreted according to the standard [17].

## 3. Results

Two rheumatologists evaluated a total of 64 patients’ radiographs. Of these patients, 40 (62.5%) were female, and 24 (37.5%) were male. Fifteen (23.4%) of the patients tested positive for HLA-B27, while 13 (20.3%) tested negative. HLAB27 was not analysed in the remaining 36 patients. The general characteristics of the patients are summarised in Table 2. In this study, we evaluated the change in scoring of the right and left SIJs over time by two rheumatologists as well as the level of agreement between their measurements. In Rheumatologist 1’s evaluations, the median change in the right SIJ’s score was 2 (interquartile range (IQR): 1–3) at the first measurement and 1 (25th–75th percentile: 1–3) at the last measurement. The Wilcoxon signed-rank test showed this decrease to be statistically significant (test statistic = 4.49, *p* < 0.001). Moderate to good agreement was found between right joint measurements (κ = 0.659, standard error = 0.056, *p* < 0.001). For the left SIJ, the median for the initial measurement was 2 (25th–75th) percentile: 1–3), and for the final measurement, it was 1 (25th–75th percentile: 1–2). The Wilcoxon signed-rank test results showed a significant decrease (test statistic = 4.69, *p* < 0.001). Moderate agreement was found between left joint measurements (κ = 0.601, standard error = 0.062, *p* < 0.001). These results indicate that the assessments of the right and left SIJs performed by Rheumatologist 1 showed significant decreases in scores over time and an acceptable level of consistency between the measurements.

For the right SIJ, Rheumatologist 2’s assessment scores were a median of 2 (25th–75th percentile: 1–2) at the initial measurement, remaining stable at a median of 1 (25th–75th percentile: 1–2) at the final measurement. The Wilcoxon signed-rank test results showed that this change was not statistically significant (test statistic = 1.06, *p* = 0.289). Moderate agreement was found between right joint measurements (κ = 0.595, standard error = 0.061, *p* < 0.001). In the left SIJ, the scores decreased from 2 (25th–75th percentile: 1–3) to 1 (25th–75th percentile: 1–2), and this decrease was statistically significant (test statistic = 2.47, *p* = 0.014). Moderate agreement was also found for the left joint measurements (κ = 0.614, standard error = 0.062, *p* < 0.001). These findings indicate that there was a significant decrease in left SIJ scores when assessed by Rheumatologist 2, while there was no significant change in the right SIJ. Moderate agreement was found for both joints.

The results show that there was a change in the scores of the right and left SIJs over time within each rheumatologist’s evaluations and that a moderate level of consistency was achieved between the measurements. The rheumatologists’ findings regarding intra-observer agreement are presented in Table 3. A statistically significant difference was found between the initial right SIJ scores of the two rheumatologists, as determined by the Wilcoxon signed-rank test (test statistic = 4.487, *p* < 0.001). This suggests that rheumatologists vary in their clinical judgement when assessing the same patients. Weighted Kappa analysis revealed moderate agreement between measurements (κ = 0.534, standard error = 0.067, *p* < 0.001). A significant difference was also found in the final right SIJ scores (test statistic = 2.828, *p* = 0.005), with good agreement (κ = 0.774, standard error = 0.050, *p* < 0.001). A significant difference was also found between the two rheumatologists’ first measurement scores for the left SIJ (test statistic = 2.837, *p* = 0.005), with moderate agreement (κ = 0.609, standard error = 0.058, *p* < 0.001). Conversely, no significant difference was found between the two rheumatologists’ final left SIJ scores (test statistic = 0, *p* = 1.000), and agreement was deemed very good (κ = 0.855, standard error = 0.039, *p* < 0.001). These results suggest that initial variations in clinical assessment between rheumatologists diminish over time, particularly in assessments of the left SIJ. The rheumatologists’ findings regarding inter-observer agreement are presented in Table 4.

**Figure 1:**
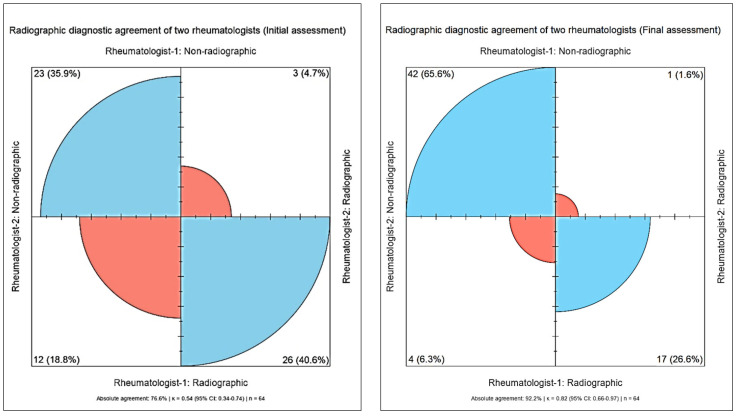
Rheumatologists’ assessment agreement on radiographic and non-radiographic diagnosis before and after clinical information. Red = disagreement; blue = agreement.

The evaluation results for the rheumatologists were also categorised as radiographic or non-radiographic. In the initial assessment, 76.6% of the assessors agreed. The kappa coefficient calculated during this evaluation was 0.54 (95% CI: 0.34–0.74), indicating a moderate level of agreement. When the distribution according to diagnostic classes was analysed, the number of cases in which both evaluators agreed on a ‘radiographic’ diagnosis was 26 (40.6%), and the number of cases in which both evaluators agreed on a ‘non-radiographic’ diagnosis was 23 (35.9%; Figure 1). In the final evaluation, the inter-rater agreement was 92.2%. The Kappa coefficient was 0.82 (95% CI: 0.66–0.97), indicating a high level of agreement. According to the final evaluation results, both rheumatologists agreed on a ‘non-radiographic’ diagnosis in 42 cases (65.6%) and a ‘radiographic’ diagnosis in 17 cases (26.6%). In other words, an increase in inter-rater diagnostic consistency was observed at the end of the evaluation process (Figure 1).

## 4. Discussion

In this study, the SIJ radiographs of 64 patients were evaluated by two rheumatologists. The scorings of the SIJs by the two rheumatologists were compared, revealing a certain degree of variation in their evaluation processes. Statistically significant differences were found between the two rheumatologists’ initial and final measurements of the right SIJ. However, the level of agreement observed improved in the final measurement. For the left SIJ, although there was a significant difference at the initial measurement, this disappeared at the final measurement, with the level of agreement reaching an excellent standard.

For a long time, the radiographic imaging of the SIJs was the primary method of identifying sacroiliitis in patients with ankylosing spondylitis (AS), allowing the visualisation of post-inflammatory structural alterations before spinal features such as syndesmophytes or vertebral fusion became evident [11]. Despite advancements in imaging, sacroiliac radiographs continue to play a central role in the classification of SpA, particularly for patients with suspected axial involvement [15]. Nonetheless, the reliability of SIJ radiography is debated, as it is often limited by significant interobserver variability [12].

A study was conducted to evaluate the effect of training on the evaluation of SIJ radiographs by radiologists and rheumatologists. The physicians evaluated the radiographs before and three months after the training. However, there was no improvement in their assessment performance after training [12]. Christiansen et al. report on the reliability of the radiographic evaluation of SIJs in a group of 104 patients with early axSpA and a symptom duration of two to twelve months. In this study, the level of agreement was found to be κ = 0.27 between two junior rheumatologists, κ = 0.34 between two senior rheumatologists and κ = 0.46 between two radiologists when the modified New York criteria were met. These data suggest that training and experience have a slight effect on inter-reader variability in the evaluation of SIJ radiographs [18]. Due to inconsistencies in the evaluation of SIJs using AP pelvic radiography, studies have been conducted to determine whether special radiographs of SIJs would produce more consistent results. These studies evaluated the SIJ view taken in a prone position, oblique views, the Ferguson view and AP lumbar radiography. However, no improvement in performance was achieved in diagnosing sacroiliitis [9,19,20,21]. In our study, we found moderate-to-substantial agreement between rheumatologists in the first blinded evaluation (right κ = 0.534, left κ = 0.609) and statistically significant differences in interpretation between rheumatologists (right *p* < 0.001, left *p* < 0.001). Our study and previous studies reveal that evaluation using SIJ radiography may not be entirely objective.

Previous studies have shown limited reliability on the part of SIJ radiography, with κ values often falling in the moderate range despite training or alternative radiographic projections. Our results extend this literature by showing that contextual clinical data can make agreement levels substantial. This raises the practical question of whether clinical information should be routinely integrated into radiographic evaluation in clinical care while maintaining central reading principles in clinical trials to minimise bias. Balancing these competing priorities remains an important challenge for the field.

A notable finding in our study is the systematic decrease in SIJ scores in three of the four assessments when clinical information was available. This pattern suggests that blinded evaluation may lead to over-reading or defensive grading in borderline cases, where uncertainty can drive readers toward assigning higher grades to avoid missing potential pathology. Once clinical features did not support a severe or highly active disease profile, the readers appeared more confident in assigning lower grades during the informed assessment. Therefore, the score reduction likely reflects a shift from caution-driven interpretation to a more clinically harmonised judgement, indicating that clinical context helps recalibrate radiographic assessment in ambiguous cases.

In a study evaluating SIJ radiographs taken at two-year intervals to assess progression, it was reported that 5.7% of patients progressed from radiographic axSpA to non-radiographic axSpA [22]. In another study examining disease progression, changes of at least one grade in one SIJ were observed in 16.8% of patients classified as progressors and in 6.3% of those classified as regressors [23]. Another study, in which the same radiographs were evaluated one day later by the same assessor, reported moderate intra-observer agreement (κ: 0.534) [24]. In our study, the intra-observer agreement ranged from moderate to substantial, and there were statistically significant (*p* < 0.001) differences between the initial and final evaluations. The different interpretations on the part of the same assessor at different times reported in our study and the other mentioned studies may be due to the insufficient reliability of the radiographic evaluation of SIJ. Additionally, the clinical information provided for the second evaluation performed in our study may have influenced the results.

Van den Berg et al. investigated the agreement between local readers (radiologists/clinicians at participating centres) and centralised readers (trained experts). The agreement between the two central readers was found to be moderate (κ = 0.56). In the same study, the agreement between the local and central readers was very similar to the inter-reader agreement between two central readers (κ = 0.55) [10]. One of the most important differences between the local reader and the central reader is that the former also has clinical information on the patient. In this study, if the central readers had clinical information, they might have reached a different level of agreement. In our study, the observers performed the initial assessment in the manner of central readers, and the final assessment was performed as local readers. Agreement increased statistically significantly at the second assessment (κ: from 0.534 to 0.774 for the right SIJ and from 0.609 to 0.855 for the left SIJ). Our study suggests that incorporating clinical information into the evaluation process may enhance inter-observer agreement. However, this improvement comes with an inherent trade-off: the informed (local reader) assessment inevitably introduces clinical anchoring because readers already know that the patient carries an SpA diagnosis. While this anchoring may bias interpretation relative to a blinded central reading, it realistically reflects how radiographs are evaluated in routine clinical care, where imaging is rarely interpreted in isolation. For this reason, the anchoring effect is not simply a methodological limitation but a necessary feature of studying real-world “local reader” behaviour. Recognising this distinction is essential when comparing informed and blinded assessments, as each represents a different but complementary aspect of clinical and research practice. We believe that the differing results between our study and that of van den Berg et al. are primarily due to differences in methodological design.

Another factor that warrants consideration is the level of reader expertise. Our study involved relatively junior rheumatologists, which may have amplified the variability in the blinded setting and the relative benefit of clinical knowledge. More experienced readers, who may rely less on contextual cues and more on subtle radiographic features, could demonstrate different patterns of change. Future studies comparing junior and senior readers would be informative in terms of clarifying whether the impact of clinical knowledge varies according to experience level.

While our findings demonstrate that access to clinical information markedly improved inter-observer agreement, this should not automatically be interpreted as a universally positive outcome. Increased consistency may partly result from ‘anchoring’ to clinical expectations, which could introduce systematic bias. For example, knowledge about HLA-B27 positivity or clinical features such as inflammatory back pain may predispose readers towards a non-radiographic classification, even in borderline radiographs. Thus, the observed gain in agreement must be balanced against the risk of over-reliance on prior assumptions, particularly in research or registry settings in which unbiased central reading is essential.

The most significant strength of our study is the fact that clinical information was presented during the second evaluation, allowing us to observe the impact of clinical data on radiological interpretation. In addition, re-evaluation by the same rheumatologists after three months allowed for the measurement of intra-observer agreement. To our knowledge, this is the first study to specifically investigate the effect of clinical information on observer agreement in SIJ radiograph interpretation. This study also has several limitations that should be acknowledged. First, it was conducted in a single centre and had a relatively small sample size, which restricts the generalisability of our findings. Second, the study population consisted only of patients already diagnosed with SpA according to the ASAS criteria, which means that the pre-test probability of the disease was already high in our cohort. In true diagnostic settings, such as among patients presenting with nonspecific chronic low back pain where SpA is only one of several possible explanations, clinicians face substantially greater uncertainty. Under these circumstances, the interaction between clinical information and radiographic judgement may differ, as readers must integrate more heterogeneous and less directive clinical cues. Therefore, the influence of clinical information observed in our study should be interpreted within the context of an already-established SpA population, and future studies including undiagnosed or early back pain cohorts are needed to determine whether similar effects occur when diagnostic uncertainty is greater. Third, the radiographic evaluations were performed by relatively junior rheumatologists. Although this reflects a realistic clinical scenario in many centres, the impact of clinical knowledge on agreement may differ among more experienced readers. Future research should compare the effects of clinical information across varying levels of reader expertise.

Because all participants in our cohort already fulfilled ASAS criteria, the study reflects interpretation in a context where the probability of SpA is high. In true diagnostic settings such as patients presenting with nonspecific chronic low back pain the pre-test probability is substantially lower. Under these circumstances, the interaction between clinical information and radiographic judegment may differ, as readers must integrate more heterogeneous and less directive clinical signals. Therefore, the effect of clinical information observed in our study should be interpreted within the constraints of an already-established SpA population, and future work including undiagnosed or early back pain cohorts is needed to determine how clinical context shapes radiographic evaluation when diagnostic uncertainty is greater.

Another important limitation is the missing data regarding HLA-B27 status in more than half of the study population (56.3%). Because HLA-B27 positivity can strongly affect both clinical suspicions and the interpretation of radiographs, this absence may have influenced the evaluation process, and it limits the strength of our conclusions. In addition, the retrospective design of the study inherently limited our ability to control patient selection and ensure the completeness of clinical data.

Future studies should investigate whether the impact of clinical information on radiographic interpretation varies across different levels of reader expertise and clinical environments. Additionally, research including larger, multi-centre cohorts and patients in earlier diagnostic stages would help clarify the generalisability of our findings and determine whether integrating clinical data can systematically enhance the reliability of SIJ radiograph assessment.

## 5. Conclusions

In conclusion, our findings indicate that the incorporation of clinical knowledge significantly reduces initial disagreement among rheumatologists. This suggests that enhancing awareness of the patient’s overall condition through clinical knowledge may improve the consistency of visual scoring.

## Figures and Tables

**Table 1:** Interpretation of kappa values and intraclass correlation coefficients.

Kappa/ICC Score	Degree of Agreement
<0.00	No agreement
0.01–0.20	Slight agreement
0.21–0.40	Fair agreement
0.41–0.60	Moderate agreement
0.61–0.80	Substantial agreement
0.81–1	Almost perfect agreement

ICC: Intraclass correlation coefficient.

**Table 2:** Descriptive characteristics of patients.

Characteristic		
**Gender**, ***n*****(%)**	Female	40 (62.5)
	Male	24 (37.5)
**HLAB27**, ***n*****(%)**	Not checked	36 (56.3)
	Negative	13 (20.3)
	Positive	15 (23.4)
**Psoriasis**, ***n*****(%)**	No	55 (85.9)
	Yes	9 (14.1)
**Low back and/or hip pain**, ***n*****(%)**	No	7 (10.9)
	Yes	57 (89.1)
**Heel pain**, ***n*****(%)**	No	49 (76.6)
	Yes	15 (23.4)
**Uveitis**, ***n*****(%)**	No	60 (93.8)
	Yes	4 (6.3)
**Inflammatory bowel disease**, ***n*****(%)**	No	62 (96.9)
	Yes	2 (3.1)
**Age**, **median (min–max)**		44 (19–62)
**Disease duration, median (min–max)**		6 (0–31)
**CRP**, **median (min–max)**		0.58 (0.3–12.6)
**ESR**, **median (min–max)**		9 (2–118)
**Duration of morning stiffness**, **median (min–max)**		30 (0–120)

HLAB27: Human leukocyte antigen B27; CRP: C-Reactive Protein; ESR: Erythrocyte Sedimentation Rate; min: Minimum; max: Maximum.

**Table 3:** Findings on intra-observer agreement for each rheumatologist.

Rheumatologist	Measurement Type	Measurement Statistics Median (25p–75p)	Wilcoxon Test Results		Weighted Kappa			
			Test Statistic	*p*-Value	κ	Standard Error	Test Statistic	*p*-Value
1	First right	2 (1–3)	4.49	<0.001	0.66	0.06	8.64	<0.001
	Final right	1 (1–3)						
	First left	2 (1–3)	4.69	<0.001	0.60	0.06	7.81	<0.001
	Final left	1 (1–2)						
2	First right	2 (1–2)	1.06	0.29	0.60	0.06	7.54	<0.001
	Final right	1 (1–2)						
	First left	2 (1–3)	2.47	0.01	0.61	0.06	7.60	<0.001
	Final left	1 (1–2)						

**Table 4:** Findings on inter-observer agreement between rheumatologists.

Measurement Type	Rheumatologist	Measurement Statistics *	Wilcoxon Test Results		Weighted Kappa				Kappa Change Statistics		
			Test Statistic	*p*-Value	κ	Standard Error	Test Statistic	*p*-Value	Difference (Kappa)	95% Confidence Interval (Difference)	*p*-Value
First right	1	2 (1–3)	4.49	<0.001	0.53	0.07	7.38	<0.001	0.24	(0.09–0.38)	<0.001
	2	2 (1–2)									
Final right	1	1 (1–3)	2.83	0.005	0.77	0.05	9.51	<0.001			
	2	1 (1–2)									
First left	1	2 (1–3)	2.84	0.005	0.61	0.06	7.59	<0.001	0.25	(0.13–0.37)	<0.001
	2	2 (1–3)									
Final left	1	1 (1–2)	0	1	0.86	0.04	10.19	<0.001			
	2	1 (1–2)									

* Values are presented as median (first quartile–third quartile)

## Data Availability

The data presented in this study are available on request from the corresponding author. The data are not publicly available due to privacy and ethical restrictions.

## Abbreviations

The following abbreviations are used in this manuscript:

SpA	Spondyloarthritis
axSpA	Axial spondyloarthritis
SIJ	Sacroiliac joint
MRI	Magnetic resonance imaging
CT	Computed tomography
CRP	C-reactive protein
HLA-B27	Human leukocyte antigen B27
ASAS	Assessment of SpondyloArthritis International Society
AP	Anteroposterior
IQR	Interquartile range
κ	Kappa coefficient
